# Developing a gender measure and examining its association with cardiovascular diseases incidence: a 28-year prospective cohort study

**DOI:** 10.1186/s12916-024-03706-3

**Published:** 2024-10-29

**Authors:** Mahée Gilbert-Ouimet, Azita Zahiriharsini, Caty Blanchette, Denis Talbot, Xavier Trudel, Alain Milot, Chantal Brisson, Peter Smith

**Affiliations:** 1 Health Sciences Department, Université du Québec À Rimouski Campus de Lévis, Lévis, Québec G6V 0A6 Canada; 2 Population Health and Optimal Health Practices Axis, Québec, Québec Canada; 3 Canada Research Chair in Sex and Gender in Occupational Health, Université du Québec À Rimouski Campus de Lévis, Lévis, Québec Canada; 4 Department of Social & Preventive Medicine, Laval University, Québec, Québec Canada; 5 Dalla Lana School of Public Health, University of Toronto, Toronto, ON Canada; 6 Institute for Work and Health, Toronto, ON Canada

**Keywords:** Sex, Characteristics related to gender, Gender measure, Cardiovascular diseases incidence, Epidemiology, Occupational health

## Abstract

**Background:**

Cardiovascular diseases (CVD) are the leading cause of morbidity and mortality worldwide. Examining gender (socio-cultural) in addition to sex (biological) is required to untangle socio-cultural characteristics contributing to inequities within or between sexes. This study aimed to develop a gender measure including four gender dimensions and examine the association between this gender measure and CVD incidence, across sexes.

**Methods:**

A cohort of 9188 white-collar workers (49.9% females) in the Quebec region was recruited in 1991–1993 and follow-up was carried out 28 years later for CVD incidence. Data collection involved a self-administered questionnaire and extraction of medical-administrative CVD incident cases. Cox proportional models allowed calculations of hazard ratios (HR) and 95% confidence intervals (CI), stratified by sex.

**Results:**

Sex and gender were partly independent, as discordances were observed in the distribution of the gender score across sexes. Among males, being in the third tertile of the gender score (indicating a higher level of characteristics traditionally ascribed to women) was associated with a 50% CVD risk increase compared to those in the first tertile (HR = 1.50; 95% CI: 1.24 to 1.82). This association persisted after adjustment for several CVD risk factors (HR = 1.42; 95% CI: 1.16 to 1.73). Conversely, no statistically significant association between the third tertile of the gender score and CVD incidence was observed in females (HR = 0.79, 95% CI: 0.60–1.05).

**Conclusions:**

The findings suggested that males within the third tertile of the gender score were more likely to develop CVD, while females with those characteristics did not exhibit an increased risk. These findings underline the necessity for clinical and population health research to integrate both sex and gender measures, to further evaluate disparities in cardiovascular health and enhance the inclusivity of prevention strategies.

**Supplementary Information:**

The online version contains supplementary material available at 10.1186/s12916-024-03706-3.

## Background

Cardiovascular diseases (CVD) are the leading cause of morbidity and mortality worldwide [1]. In North America, CVD are the costliest disease annually. They recently resulted in an estimated cost of $21.2 billion in Canada (2019) [2], and $239.9 billion in the USA (2018–2019) [3]. Worldwide, CVD incidence is increasing due to the rapid aging of the population [4]. While the incidence of CVD is higher in middle-aged males than females, aging females have a similar level of risk once the estrogen protection diminishes due to menopause [5], after which they face poorer CVD outcomes compared to males [6, 7]. Moreover, the World Health Organization (WHO) showed an increase of age-standardized CVD death rates in females aged 35–74 years in Canada and the USA in 2017 [8]. Despite these statistics and disparities, two-thirds of global heart disease research focuses on males [9, 10].

Further examinations of sex differences in the development of CVD are required to improve prevention and achieve better equity in health [11]. However, in order to move beyond simple comparisons of male and female differences, examining gender (socio-cultural) in addition to sex (biological) is also required. Sex and gender, while different concepts, are inextricably linked. Both are recognized determinants of health [12]. Sex refers to a set of biological attributes. Gender, on the other hand, is neither something a person is born with nor something a person has, but rather is related to the current social context and a person’s roles, expectations, relationships, and behaviors within that context [13]. It is important to note that gender is context-dependent (time and place) and evolves in time, as it is culturally based and historically specific [14].

The concept of gender has been defined according to four interrelated dimensions [15]: gender roles and expectations (e.g., norms and expectations typically associated with gender), gender identity (e.g., behaviors, expressions, and perception of oneself gender and others), gender relationships (e.g., how individuals act or are treated based on their gender), and “institutionalized gender” (e.g., systemic differences in power and influence that are gender-based) [15]. Considering gender according to this definition is particularly challenging in studies relying on secondary data, which rarely include a direct measure of gender, as it is generally the case in epidemiological cohorts destined to improve CVD prevention [16, 17]. The creation of measures assembling variables related to gender has been suggested and attempted. Examples of measures include work by Pelletier et al. [18], which showed that sex and gender were partly independent predictors of recurrent acute coronary syndrome [19]. This measure however includes several variables that are generally not measured in existing epidemiological cohorts and is therefore hard to export. Another example is the work of Lacasse et al. [20] using data from the Canadian Community Health Survey. The Lacasse gender index overcomes this limitation. However, it is not restricted to gender, as other dimensions of intersectionality (i.e., other factors of discrimination) are included, namely race, citizenship, and sexual orientation. Also, the measure includes household-level variables, which might contribute to misclassifying individuals based on the characteristics of other members of the household. The current study proposes a new gender measure relying on secondary data, which surpasses the main limitations of the previous measures.

To our knowledge, only the gender measure derived by Pelletier et al. [18] was examined in relation to CVD outcomes [19]. This study suggested that characteristics traditionally ascribed to women might be deleterious to cardiovascular health (non-statistically significant), while female sex might not be [19]. However, the limited sample size of this previous study (273 females and 636 males) combined with a low mortality rate over the 12-month follow-up led to low statistical power, which may explain the imprecise and non-significant findings reported. The current study overcomes this limitation by examining the association between our new gender measure and the 28-year incidence of CVD among thousands of participants.

The objectives of this study are twofold:

1. To develop a gender measure including all four recommended gender dimensions, comprising a wide range of variables that are, or could be, routinely measured in population health surveys.
2. To examine the association between this gender measure and CVD incidence over 28 years, across sexes.

## Methods

### Study design and procedure

**Fig. 1:**
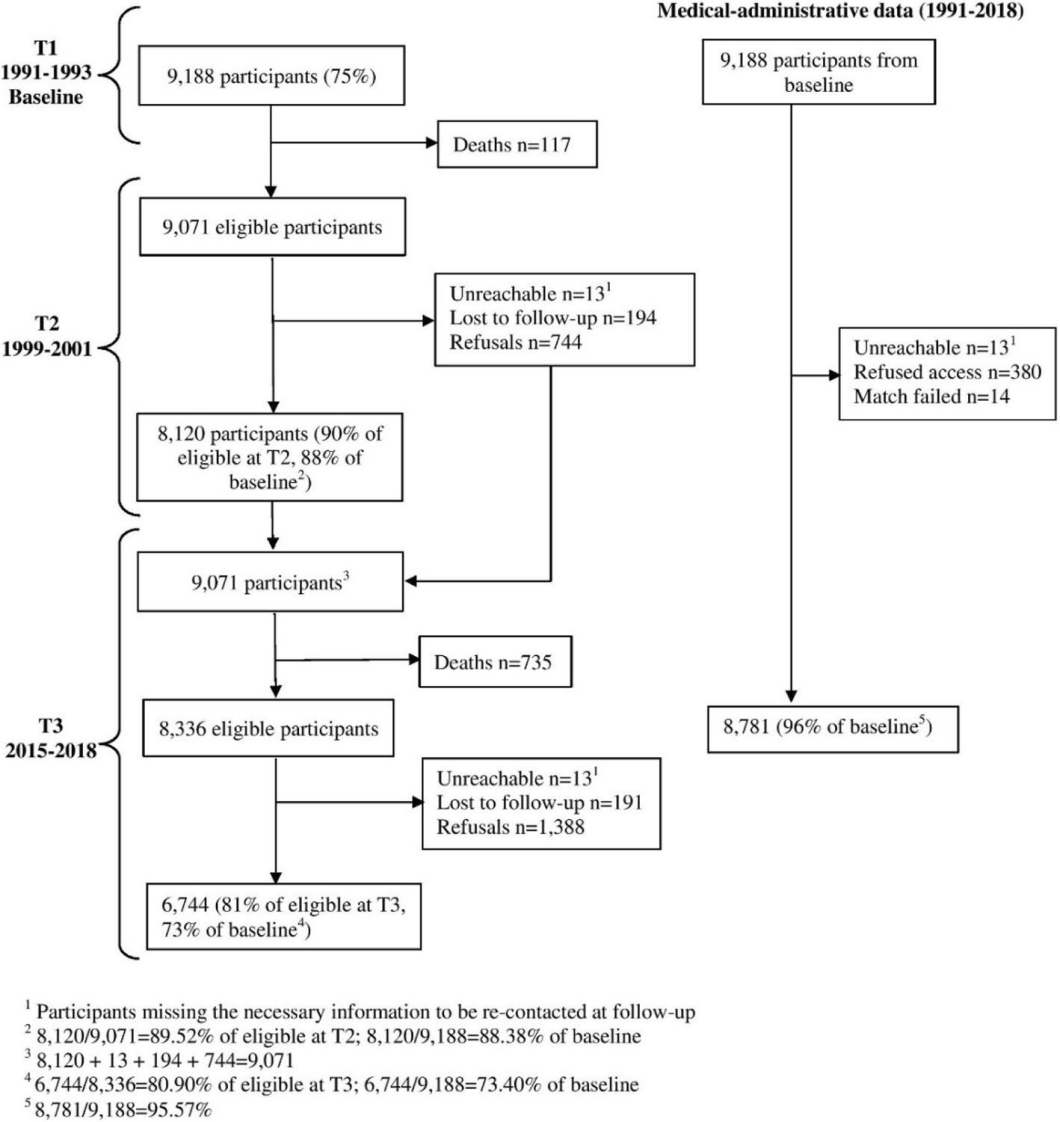
Flowchart of the PROspective Quebec (PROQ) Study on Work and Health. At baseline, 9188 subjects (75%) agreed to participate. At the 8-year follow-up, 8120 subjects participated (88% of the initial cohort). At the 24-year follow-up, 6744 subjects participated (73% of the initial cohort) in the data collection. A total of 8781 participants (96%) consented to the medical-administrative data collection and for whom the match has been made successfully at the 28-year follow-up

The study drew upon the data from the PROspective Quebec Study (PROQ-Study) on Work and Health, an established prospective cohort of 9188 white-collar females (49.9%) and males recruited in 1991–1993 and followed on average for 24 years (2015–2018) [21]. At baseline, the study population consisted of workers from 19 public and semi-public organizations in the Quebec City region, Canada. At baseline, 9188 subjects (75%) agreed to participate, including 10.4% managers, 34.1% professionals, 52.4% technicians and office workers, and 3.2% held other occupations. They were aged on average 40 years (min–max: 19–72 years). A total of 8781 participants (96%) consented to the medical-administrative data collection and for whom the match has been made successfully at the 28-year follow-up (Fig. 1).

### Data collection

Workers completed a self-administered questionnaire on demographic and socioeconomic characteristics, cardiovascular risk factors, characteristics of work, personality traits, and family load and social life. Trained personnel also measured weight, height, waist circumference, and blood pressure. At the end of the follow-up, CVD events were retrospectively extracted from medical-administrative databases for the period from January 1, 1991, to December 31, 2018.

### Sex variable

Sex was defined using a single question asking whether the participant is female or male at baseline.

### Gender measure

Several variables related to gender were measured in the PROQ-Study, covering the four interrelated gender dimensions developed by Johnson et al. [15] and recommended by the Canadian Institutes of Health Research [15]. These variables are hypothesized to be related to gender in Canada as they tend to be traditionally different in women and men (i.e., culturally and historically different). Table 1 presents these variables, their categorizations, the instruments used to measure them, the gender dimensions they were grouped under, and whether they reflect higher level of characteristics traditionally ascribed to women and men.

**Table 1:** Variables related to gender considered for the development of the gender measure and instruments used, across the four recommended gender dimensions

Gender dimensions and variables	Instruments used	Main categorization applied	Characteristics traditionally ascribed to men (M) and/or women (W)
1- **Gender role and expectations**			
Occupational position	A single question: What is your current employment status?	In five categories: (1) office workers,( 2) technicians, (3) professional workers, (4) managers, and (5) others	M: managers, technicians. W: office workers
Work time (per week)	Two questions: - How many hours per week do you work for your organization (main paid job) [22]?	In four categories: (1) 10–20 h/week, (2) 21–34 h/week, (3) 35–40 h/week, and (4) ≥ 41 h/week	W: ≥ 41 h/week in main job W: < 10 h/week in extra work
	- How many hours per week do you work in another paid job or study (extra working hours)?	In four categories: (1) none,( 2) < 10 h/week, (3) 10–20 h/week, and (4) ≥ 21 h/week	
Family load	Combining the number of children and domestic workload according to the algorithm used by Brisson et al. [23]	In three categories: (1) low, (2) moderate, and (3) high	W: high family load
Physical job demands	A single question examining the physical aspects of respondents’ job (to best describe the work they usually do)	In four categories: (1) sit or stand during the day (unexposed), (2) move around during the day (low), (3) lift or carry light loads (moderate), and (4) work hard physically (high)	M: high physical job demands
Two psychosocial stressors at work: psychological demands and job control	18 items of Karasek’s validated self-administered questionnaire [24–26]: - High psychological demands (ex. workload, interruptions, and time constraints) (9 items) - Low job control (ex. decision authority, and skill discretion) (9 items)	- Dichotomized: (1) unexposed, and (2) exposed (≥ 24) - Dichotomized: (1) unexposed, and (2) exposed (≤ 72)	M: high psychological demands M and W: low job control
2- **Gender identity**			
Anger	Items used in the Framingham heart study (Anger-In, keeping anger to oneself; Anger-Out, taking anger out on others; and Anger-Discuss, talking with a friend or relative) (7 items) [27]	In three categories: (1) low, (2) moderate, and (3) high	M: Anger-In, Anger-Out W: Anger-Discuss
Cynicism and hostility	The Cook-Medley Hostility scale (13 items) [28]	In three categories: (1) low, (2) moderate, and (3) high	M: high cynicism and hostility
3- **Gender relationships**			
Marital status	A single question: Do you live with a spouse?	Dichotomized: (1) no (single), and (2) yes (married/in a relationship)	W: married/in a relationship
Social support outside from work	The social support scale consisted of 3 items [29]: Low social support outside from work	Dichotomized: (1) unexposed, and (2) exposed	M: low social support outside work
Another psychosocial stressor at work: social support at work	5 items from MONICA project [30]: Low social support at work	Dichotomized: (1) unexposed, and (2) exposed (≥ 11)	M and W: low social support at work
4- **Institutionalized gender**			
Education level	A single question: What is the highest level of education you have completed?	In three categories: (1) secondary, (2) college, and (3) university	M: higher education level (university)

A gender score was calculated to categorize participants according to the level of characteristics traditionally ascribed to women and men. The score described the probability of being female (between 0 and 1) for each respondent. Thus, this gender measure was visualized as a continuum of higher levels of characteristics traditionally ascribed to men (gender scores toward 0) to higher levels of characteristics traditionally ascribed to women (gender scores toward 1).

### Cardiovascular diseases incidence

CVD events included ischemic heart disease (IHD) and stroke. For the 28-year of follow-up, incident cases of CVD were retrieved from medical-administrative databases with recourse to validated algorithms for IHD (sensitivity: 77.0%; specificity: 97.5%; positive predictive value: 75.3%) [31], and stroke (sensitivity: 60.2%; specificity: 99.2%; positive predictive value: 62.0%) [32]. Diagnostic (*International Classification of Disease* (ICD)-9 and 10 codes) and procedure codes (Canadian Classification of Diagnostic, Therapeutic, and Surgical Procedures (CCP) and Canadian Classification of Health Interventions (CCI)) were extracted from two public healthcare administrative databases to identify CVD cases among the PROQ Study participants: (1) the RAMQ ([Régie de l’Assurance Maladie du Québec] healthcare insurance board) database, which contains information on physician claims, covering various types of medical visits, namely inpatient, outpatient, intensive care, and emergency visits and (2) the MED‐ECHO database (Maintenance et exploitation des données pour l’étude de la clientèle hospitalière), which compiles data pertaining to hospital stays in Quebec, covering both general and specialized care settings. Furthermore, national death registry databases were searched and causes of death (ICD-9 and ICD-10) were extracted [33]. The incidence date of CVD was defined as the first date when one of the IHD or stroke was identified by their respective algorithms.

IHD codes were as follows: ICD-9 410–414; ICD-10 I20–I25; CCP: 48.02, 48.03, 48.09, 481, and CCI: 1.IJ.50, 1.IJ.57.GQ, 1.IJ.76 [31]. Stroke codes were: ICD-9 362.3, 430, 431, 432, 434.X, 435.X, 436; ICD-10 I60.X, I61.X, I63.X (excluding I63.6), I64, H34.0, H34.1, and G45.X (excluding G45.4) [32]. Participants with myocardial infarction, angina, acute and chronic coronary syndrome, and those who have had a percutaneous coronary intervention, coronary artery bypass or angioplasty, and with stroke, including transient ischemic attack, were identified during the period of selection if they met one of the following criteria: (1) had at least one hospitalization with a diagnosis or procedure code for IHD or stroke or (2) had two or more physician claims with diagnosis for IHD or stroke within a 1-year period, or (3) had IHD or stroke listed as the primary cause of death [31, 32].

### Covariates

CVD risk factors were measured as potential covariates at baseline. Smoking [34], alcohol consumption [35], and physical activity [36] were assessed using validated self-administered questionnaires. Family history of CVD, diabetes, cholesterol, and antihypertensive medication were also self-administered. Weight and height for body mass index (BMI), and waist and hip circumferences for waist-hip ratio (WHR), were measured by trained personnel following a validated protocol [37]. Blood pressure was taken in both arms after 5 min of rest by trained nurses following the American Heart Association protocol [38]. An average of three blood pressure measurements were obtained with a 1–2 min interval.

### Statistical analyses

The gender measure developed as our first study’s objective was developed following the exportable analytical steps of Pelletier et al. [18], with a sole difference in the approach chosen for selecting the variables related to gender (steps 0 and 1). These steps are described hereafter, along with indications to ease replication. It is also noteworthy that our gender measure was the first to be evaluated in relation to the incidence of cardiovascular disease.

- **0. Selecting variables related to gender based on the literature:** Before proceeding with the analytical steps, variables reflecting characteristics that tend to be culturally and historically ascribed to men or women should be selected. For comparability, variables routinely measured in existing databases within the studied field of research may be chosen. In the current study, a total of 16 variables were initially selected (Table 1).
- **1. Determining which variables are related to gender based on data:** This step involves evaluating whether the variables hypothesized to be associated with gender, as identified in step 0, are correlated with sex. Correlation with sex was applied because gender is a multidimensional construct linking gender identity, gender expression, as well as social and cultural norms and expectations associated with sex [39]. This approach has also been chosen in previous studies to develop gender measures (e.g., 18, 20, 32), which used a similar definition of variables related to of gender (those differing between sexes) with the shared the objective to create measures that distinguish men and women based on psychosocial characteristics. An exploratory logistic regression was conducted with sex as the dependent variable and baseline variables related to gender as the independent variables. Variables associated with sex were then identified using the LASSO selection method, with all predictors included in the model. Coefficients of variables not associated with sex were shrunk to zero while estimating the coefficients of others, as the LASSO method does not inherently remove variables from the final model. This method allowed for variables that were deemed non-significant or redundant to be removed from the subsequent steps, resulting in a more parsimonious gender measure optimizing both predictive accuracy and interpretability. This method, known for its robustness, offers higher prediction accuracy compared to alternative regression models used in similar studies [40, 41], such as Pelletier et al. [18]. The hyper-parameter of the LASSO was determined by optimizing the Bayesian Information Criterion.
- **2. Confirming the gender measure:** This step consists of testing the gender measure accuracy to predict sex. Predictive accuracy was evaluated using the c-statistic [42]. Retained variables predicted sex more effectively if the c-statistic surpassed 0.5.
- **3. Calculating the gender score:** The gender score is then calculated by aggregating the retained variables. In the final logistic regression, coefficient estimates of variables related to gender were used to derive a continuous gender score ranging from 0 to 1 (which correspond to predicted values given the binary outcome).
- **4. Grouping individuals:** Study participants are then divided into three groups based on their gender score, to reflect the non-binary nature of gender. Tertile cut-offs based on the gender score were applied: tertile 1 indicated a higher level of characteristics traditionally ascribed to men (predicted values of 0.29385 or lower), tertile 2 reflected a mix of characteristics (predicted values between 0.29386 and 0.67886), and tertile 3 indicated a higher level of characteristics traditionally ascribed to women (predicted values of 0.67887 and above). For instance, consider a participant with a gender score of 0.35. According to the tertile cut-offs, this individual falls into the second tertile when they have a balanced mix of characteristics traditionally ascribed to both women and men based on the study’s variables and scoring method.
- For objective 2, the associations between gender tertiles at baseline and the 28-year CVD incidence across sexes were assessed with hazard ratios (HRs) using the Cox proportional hazards regression model with chronological age as the time scale [43] and delayed entry at the age at the study baseline. Survival time for each participant was calculated from the date of birth up until (1) CVD diagnosis, (2) competing risk (i.e., death), or (3) the end of follow-up, whichever came first. Statistical adjustment was applied for self-reported sex-related variables for females, including menopausal status, use of oral contraceptives, and hormone replacement therapy. Further adjustment was made for CVD risk factors at baseline as potential covariates.

### Sensitivity analyses

Three sensitivity analyses were performed to assess the robustness of the findings. In the first two, alternative versions of the gender measure were created excluding variables a priori hypothesized as potentially driving a large portion of the association with CVD. These variables were respectively (i) psychosocial stressors at work (PSWs), i.e., psychological demands, job control, and social support at work, as well as working hours, and (ii) personality traits, i.e., anger, hostility, and cynicism. These two versions of gender measures and associated analyses were compared with the complete version including all variables related to gender from Table 1. Finally, survival analyses including only ischemic heart diseases (i.e., excluding strokes) were conducted, as this CVD subtype was the most prevalent in our sample and might have a partly distinct etiology than stroke.

## Results

### Variables related to gender in the PROQ-Study

The analysis of the 16 variables related to gender (Table 1) revealed that only hostility was excluded through the initial LASSO selection method, and 15 variables were included in the gender measure. In Table 2, odds ratios (ORs) and 95% confidence intervals (CIs) show the association between these variables and female sex in the final multivariable logistical model. All variables, except hostility, were positively or negatively associated with the probability of being female, with similar strength of associations observed for most variables. Characteristics hypothesized to be related to masculine gender, namely marital status (being married), physical job demands (low to high), anger-in (high), cynicism (moderate and high), low social support outside from work, level of education (college and university), and occupational position (all but office workers the reference) were negatively associated with female sex. Conversely, characteristics hypothesized they reflect feminine gender, which were working hours in the main paid job (21–34 h/week), family load (moderate and high), high psychological demands, low job control, anger-discuss (moderate and high), and low social support at work, were positively associated with female sex.

**Table 2:** Associations between the variables related to gender at baseline (1991–1993) and sex, modeling the probability of being a female (*N* = 8429)

**Gender-related variables**		**OR (95% CI)**	p*
Marital status	Single	(1.00) (–)	< .0001
	Married/in a relationship	**0.55 (0.47–0.64)**	
Working hours in main paid job	10–20 h/w	2.01 (0.90–4.50)	< .0001
	21–34 h/w	**2.03 (1.58–2.60)**	
	35–40 h/w	(1.00) (–)	
	≥ 41 h/w	0.94 (0.75–1.18)	
Hours working overtime	None	(1.00) (–)	0.0677
	< 10 h/w	**0.83 (0.72–0.96)**	
	10–20 h/w	0.90 (0.72–1.12)	
	≥ 21 h/w	0.86 (0.56–1.35)	
Family load	Low	(1.00) (–)	< .0001
	Moderate	**1.27 (1.08–1.49)**	
	High	**1.49 (1.27–1.74)**	
Physical job demands	Unexposed	(1.00) (–)	< .0001
	Low	**0.82 (0.73–0.93)**	
	Moderate	**0.25 (0.18–0.35)**	
	High	**0.25 (0.11–0.59)**	
High psychological demands	Unexposed	(1·00) (–)	< .0001
	Exposed	**1.44 (1.29–1.62)**	
Low job control	Unexposed	(1·00) (–)	0.0003
	Exposed	**1.24 (1.11–1.40)**	
Anger-In	Low	(1.00) (–)	0.0024
	Moderate	0.87 (0.76–1.00)	
	High	**0.80 (0.70–0.92)**	
Anger-Out	Low	(1.00) (–)	0.2331
	Moderate	1.13 (1.00–1.29)	
	High	1.01 (0.87–1.17)	
Anger-Discuss	Low	(1.00) (–)	< .0001
	Moderate	**1.52 (1.32–1.76)**	
	High	**2.70 (2.33–3.12)**	
Cynicism	Low	(1.00) (–)	< .0001
	Moderate	**0.88 (0.78–0.99)**	
	High	**0.65 (0.56–0.76)**	
Low social support outside from work	Unexposed	(1.00) (–)	< .0001
	Exposed	**0.52 (0.46–0.58)**	
Low social support at work	Unexposed	(1.00) (–)	< .0001
	Exposed	**1.30 (1.16–1.44)**	
Level of education	Secondary	(1.00) (–)	< .0001
	College	**0.62 (0.53–0.72)**	
	University	**0.50 (0.42–0.60)**	
Occupational position	Office workers	(1.00) (–)	< .0001
	Technicians	**0.21 (0.18–0.24)**	
	Professional workers	**0.11 (0.09–0.14)**	
	Managers	**0.05 (0.04–0.07)**	
	Others	**0.28 (0.21–0.37)**	

Bold values denote statistical significance (*p* < 0.05)

*p for global test using the Wald chi-square statistic (Type 3 Analysis of Effects)

### Predictive accuracy

The estimation of predictive accuracy using the c-statistic yielded a significant value of 0.831 (95% CI: 0.823–0.840), indicating that our model elucidated sex markedly better than chance.

### Gender score distribution in the PROQ-Study

**Fig. 2:**
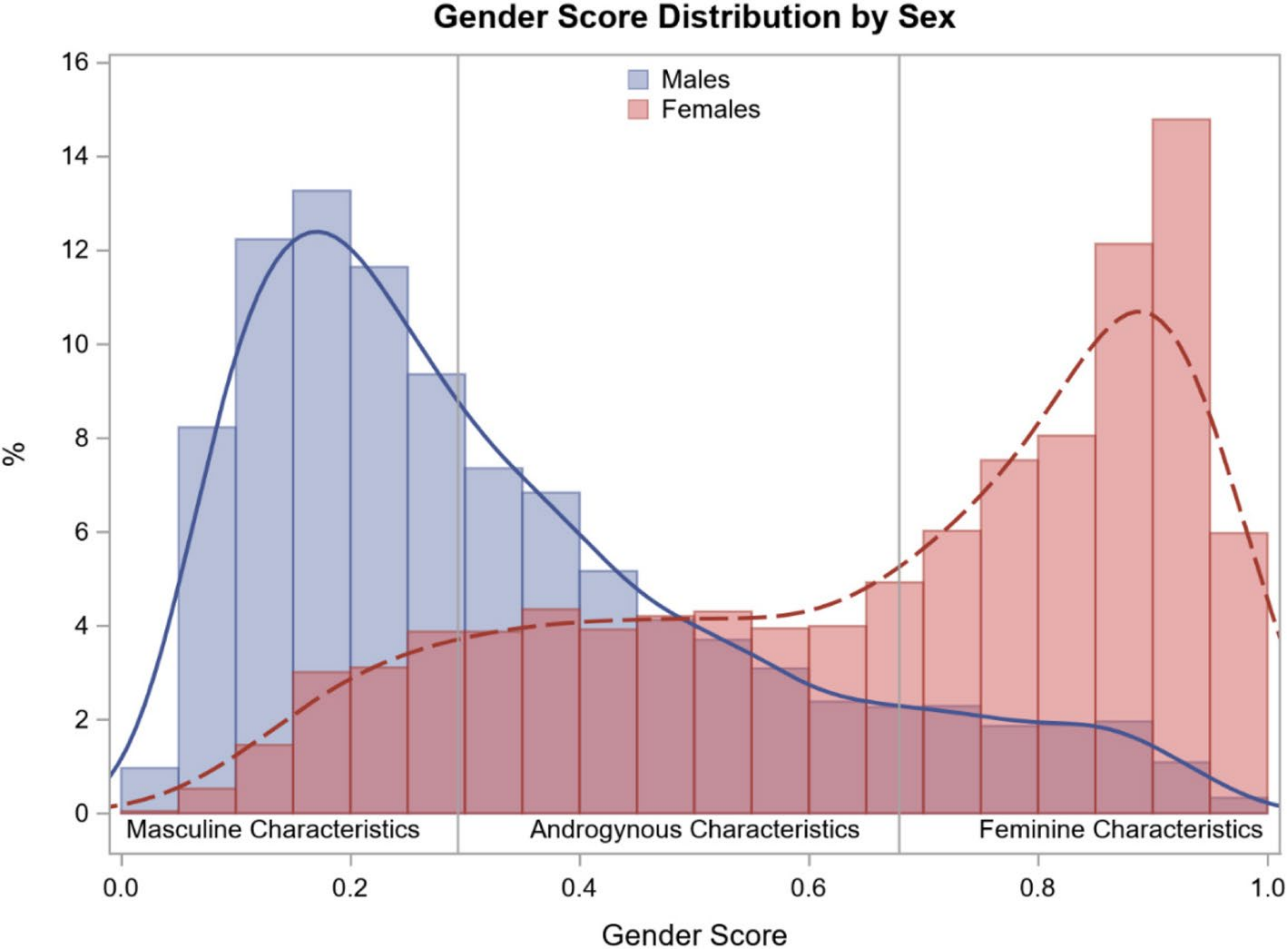
Gender score distribution in men and women from the PROQ-Study. The figure represents the discordances in the distribution of the gender score across sexes. There were males and females at all parts of the distribution with similar gender scores

Sex and gender were partly independent, as discordances were observed in the distribution of the gender score across sexes. Figure 2 highlights the presence of males and females across all sections of the distribution, displaying similar gender scores. When applying gender tertiles, scores varied from 0.01298 to 0.29385 for predominantly masculine characteristics, from 0.29386 to 0.67886 for androgynous characteristics, and from 0.67887 to 0.98956 for feminine characteristics.

### Description of the participant characteristics

Participants’ baseline characteristics are presented in Table 3 across the gender tertiles. Participants with characteristics traditionally ascribed to feminine (tertile 3) were more likely to be females (84.5%), to smoke (29.8%), to have a sedentary lifestyle (47.8%), and family history of CVD (34.7%). However, participants in this tertile were less likely to be married (64.7%), to have high-risk WHR (18.2%), high-risk alcohol consumption (3.0%), cholesterol (12.3%), and hypertension (8.9%). Participants’ characteristics are presented across sexes in Table S1. In general, the masculinity-male versus femininity-female results seem to point in the same direction.

**Table 3:** Participant characteristics according to the tertiles of the gender score, at baseline (1991–1993)

	**Tertile 1 Masculine** **characteristics (*N* = 2810)**	**Tertile 2 Masculine and feminine** **characteristics (*N* = 2809)**	**Tertile 3 Feminine** **characteristics** **(*N* = 2810)**	p*
*N* (%) or mean (SD)				
**Demographic characteristics**				
Age, years, mean (SD)	42.0 (8.7)	38.6 (8.4)	39.1 (8.2)	< .0001
Female	492 (17.5)	1,320 (47.0)	2,374 (84.5)	< .0001
Married/in a relationship	2,402 (85.5)	1,962 (69.8)	1,818 (64.7)	< .0001
CVD risk factors**				
Obesity	273 (9.7)	238 (8.5)	260 (9.3)	0.2827
High-risk WHR	1,544 (55.4)	1,027 (37.0)	503 (18.2)	< .0001
Current smoking	514 (18.4)	602 (21.6)	831 (29.8)	< .0001
High-risk alcohol consumption	176 (6.3)	110 (3.9)	85 (3.0)	< .0001
Low physical activity	1,049 (37.4)	1,130 (40.3)	1,339 (47.8)	< .0001
Family history of CVD	821 (29.5)	795 (28.6)	963 (34.7)	< .0001
Diabetes	63 (2.3)	52 (1.9)	56 (2.0)	0.5696
Cholesterol	577 (20.7)	391 (14.0)	343 (12.3)	< .0001
Hypertension status	438 (15.6)	326 (11.6)	249 (8.9)	< .0001
Antihypertensive medication	86 (3.1)	63 (2.3)	82 (2.9)	0.1324
Menopause***	40 (8.1)	88 (6.7)	237 (10.0)	0.0025
Use of oral contraceptives***	412 (83.9)	1,100 (84.4)	1,929 (82.1)	0.1747
Hormone replacement therapy***	48 (9.8)	102 (7.8)	252 (10.7)	0.0162

^*^p for *F*-test (for age continuous) and chi-square test (for all other categorical variables)

^**^Missing values are present for these different variables

^***﻿^Only for females

### Association between the gender score and CVD incidence across sexes

A total of 1086 (27.5%) and 519 (13.4%) incident CVD cases were recorded among males and females, respectively. Males contributed to 89,800-person years and females to 94,729-person years resulting in a crude cumulative CVD incidence of 12.1 and 5.5 per 1000-person years, respectively.

The association between the gender score and the 28-year CVD incidence differed by sex (*p* = 0.0015 for interaction effect in the multivariable model), so results are presented separately for males and females. Among males, being in the third tertile of the gender score (indicating a higher level of characteristics traditionally ascribed to women) was associated with a 50% CVD risk increase compared to those in the first tertile (HR = 1.50; 95% CI: 1.24 to 1.82) (Table 4). This association persisted after adjustment for CVD risk factors (HR = 1.42; 95% CI: 1.16 to 1.73). Among females, being in the third tertile was associated with a decreased CVD risk in the adjusted model, although statistical significance was not reached (HR = 0.79, 95% CI: 0.60–1.05). In addition, female sex had a protective association with CVD incidence (HR = 0.65, 95% CI: 0.56–0.77) (Table S2).

**Table 4:** Associations between gender tertiles at baseline (1991–1993) and the 28-year CVD incidence, stratified by sex (*N* = 7821)

	Number of participants	Number of events	HR (95% CI)^a^	HR (95% CI)^b^
**Males**	3956	1086		
Tertile 1	2183	623	1·00 (–)	1·00 (–)
Tertile 2	1390	332	0.99 (0.87–1.14)	1.00 (0.87–1.15)
Tertile 3	383	131	**1.50 (1.24–1.82)**	**1.42 (1.16–1.73)**
**Females**	3865	519		
Tertile 1	466	67	1·00 (–)	1·00 (–)
Tertile 2	1230	156	0.94 (0.71–1.26)	0.91 (0.67–1.23)
Tertile 3	2169	296	0.91 (0.69–1.18)	0.79 (0.60–1.05)

Bold values denote statistical significance (*p* < 0.05)

^a^Age adjusted through time scale and further adjusted for menopausal status, use of oral contraceptives, and hormone replacement therapy at baseline for females. *N* = 3811 for females because of missing values for these different variables

^b^Further adjusted for BMI (kg/m^2^): BMI (underweight/normal (< 25), overweight (25–29.9), and (3) obesity (≥ 30)); WHR: (1) low risk (< 0.90 in men and < 0.85 in women), and (2) high risk (≥ 0.90 in men and ≥ 0.85 in women); smoking: (1) non-smoker, (2) former smoker, and (3) current smoker (occasional + regular)); alcohol consumption: (1) abstinent (0 consumption/week), (2) low risk (≤ 15 consumptions/week for men and ≤ 10 for women), and (3) high risk (> 15 consumptions/week for men and > 10 for women)); physical activity: (1) active (≥ 3 weekly), (2) moderate (1 or 2 weekly), and (3) low (< 1 weekly)); family history of CVD: (1) no, and (2) yes), diabetes (1) no, and (2) yes; cholesterol: (1) no, and (2) yes; hypertension status: a person was considered hypertensive if they had a blood pressure (SBP) ≥ 140 and/or diastolic blood pressure (DBP) ≥ 90; (1) no, and (2) yes; antihypertensive medication (1) no, and (2) yes) at baseline. *N* = 3778 for males and *N* = 3583 for females because of missing values for these different variables

### Sensitivity analyses

Two alternative gender measures were computed excluding respectively (i) psychosocial stressors at work and working hours or (ii) personality traits, to examine the robustness of the associations with CVD with and without these variables as part of the gender measure. For both alternative gender measures, associations were similar to those observed with the complete gender measure. After exclusion of psychosocial stressors at work and working hours or personality traits (adjusted for CVD risk factors), males within the third tertile of the gender score still had a greater risk of CVD (30% and 42%, respectively) compared with males in the first tertile, while there was no increased risk in females (Tables S3 and S4). The sensitivity analysis considering only ischemic health disease incidence also yielded similar results.

## Discussion

Recent policies from the US, Canada, and the European Union underscored the importance of incorporating both sex and gender into health research to ensure scientific rigor and to enhance inclusivity and equity in educational and prevention efforts [44]. The present study proposed the development of a comprehensive gender measure through a rigorous and exportable process assembling characteristics related to gender commonly measured in epidemiological, as well as occupational and public health cohorts. Using such gender measure allows measuring gender when you do not have a gender measure, capturing different gender dimensions, and moving beyond female-male (sex) comparisons. In our sample of thousands of workers, discordances were observed in the distribution of the gender score across sexes (ex. about one in six participants with a feminine gender score were males), highlighting the complementarity between gender measure and sex. Among males, being in the third tertile of the gender score was associated with an increased incidence of CVD, and this association was robust to adjustment for CVD risk factors. Such an increased risk was not observed among females. To our knowledge, this study was the first to examine the association between such a gender measure and CVD across sexes, i.e., separately for females and males. Our findings suggest that primary prevention strategies aimed at reducing CVD may need to incorporate the consideration of characteristics related to gender in addition to sex, in particular among males.

In the current study, a new gender measure was developed overcoming the main limitations of the previous measures relying on secondary data, by covering all four recommended gender dimensions and including a wide range of individual characteristics. Several variables included in the current gender measure were consistent with the variables retained in the previous gender measures, such as marital status [20], working hours, education, occupation [20, 45], family load [18, 20, 45, 46], job strain [46], and social support both within and outside the workplace [46]. When a primary data collection of gender is unfeasible, examining the role of gender through such a measure might enhance the understanding of health determinants [46, 47] and provide a basis to uncover explanations for male and female differences in health conditions [18, 19].

If only sex had been examined in our cohort, the adverse effect observed in males within the third tertile of the gender score would have gone unnoticed. While the association between characteristics traditionally ascribed to women and poorer CVD outcomes has previously been reported [19], very little is known about this association across sexes. A potential explanation for the increased CVD risk in males of the third tertile of the gender score could be the stress arising from assuming multiple work and family roles. Males with multiple roles may tend to feel more pressured to perform at work than females, as social contributions such as caring for relatives and communal roles are still more socially expected to be feminine responsibilities. A study led by US volunteers reported that males who requested a family leave or flexible hours due to family responsibilities were rated as lower on organizational commitment, ineligible for rewards, and suffered from femininity stigma [48]. It is also noteworthy that males with other status inconsistencies, such as a discordance between education level and occupation status, were also previously shown to be at increased risk of CVD outcomes [49].

Another potential explanation for the increased CVD risk observed among males within the third tertile of the gender score might lie in the underlying intersectionality between variables of our gender measure. Some gendered characteristics included in our measure were socioeconomic indicators known to reinforce health inequalities [1, 50, 51]. We assigned as “traditionally ascribed to women” lower levels of education, occupation, and income based on previous studies [15, 52], including Canadian data from the early years of the current study (1990s) [52]. The estimates from our regression model (the predicted probability) confirmed these gendered assignments. Education, occupation, and income have been consistently associated with CVD outcomes in the literature [50, 53, 54]. Sex differences were previously observed, as lower socioeconomic status was more strongly associated with unhealthy diet and lifestyle as well as less optimal and specialized health care and self-care in males than females [55]. In addition, a systematic review and meta‑analysis revealed an increased risk of CVD mortality in males with low socioeconomic status than females with low socioeconomic status, possibly because of delayed or lack of CVD care and treatment [56].

To further understand the increased CVD risk in males within the third tertile of the gender score, sensitivity analyses exploring whether specific characteristics of the gender measure held a particular importance in the association were performed. When examined separately, four of the 15 gender characteristics included in the measure were significantly associated with CVD in males; education level and occupational position, job control, and personality traits (Table S5). In contrast, among females, the observed associations were less pronounced (Table S5). Three alternative gender measures were built to examine results robustness when excluding these variables. For the first one, excluding education and occupation, results pointed in the same direction as those of the main analyses, although hazard ratios were attenuated and became borderline significant. Considering the determining role of socioeconomic indicators in the CVD incidence, such attenuation was expected. In addition, and as described previously, the two other gender measures excluded either psychosocial stressors at work or personality traits and yielded similar results to those obtained using the complete gender measure. Hence, these four variables appear to be particularly important ingredients of the gender measure for males but, consistently with a holistic approach, examining them as part of a combination of gendered characteristics might be preferable to capture the association with CVD.

In females, the fact that being in the third tertile of the gender score pointed towards a protective effect against developing CVD was unexpected. However, it is plausible. In females, being a mother and taking care of the family is known to improve psychological well‐being and reduce physical signs of stress by providing satisfaction and self‐rewards [57], which may lower CVD risk. Also, having to deal with a high level of family responsibilities does not systematically imply that work and personal spheres are negatively interfering. A consistency between characteristics related to sex and gender, thus, may contribute to the lower risk of CVD incidence among females. This hypothesis was further explored through post-hoc analysis applying a finer categorization of the gender score into sextiles (six approximately balanced groups) and examining its association with CVD incidence (Table S6) to explore the potential presence of a gradient (dose–response). Similar results were however obtained as with the main analysis using tertiles. Estimates from both the 5th and 6th categories were nearly identical (HR5th = 0.78 (95% CI: 0.50–1.23) and HR6th = 0.79 (95% CI: 0.50–1.24) compared to that of the highest tertile (HR = 0.79 (95% CI: 0.60–1.05)), with no significant linear trend observed (*p* = 0.1382). Our findings among females should nevertheless be interpreted with caution, and should not be generalized without further replication. The approach of selecting variables based on their ability to predict sex in our gender measure can be debated. As previously mentioned, after selecting gender characteristics based on literature, variables ‘objectively’ associated with the reality of being biologically female or male in our cohort were chosen, i.e., at a given time and place, similarly to what was done in existing published gender measures based on secondary data [18, 20, 58]. Criticism to this approach includes the limited generalizability of such measure and the difficulty in examining the differences in the relationship between gender and sex over time or location. An alternative approach consists of selecting a priori the individual characteristics related to gender based on the literature only, independently of how they relate to being a female or a male in a given sample. In post hoc analyses, our gender measure was rebuilt using this approach, by including all 16 variables that were initially selected based on the literature. Associations between this gender measure and CVD incidence were almost identical (Table S7).

### Study strengths and limitations

The present study relied on a 28-year follow-up cohort of 9188 female (49.9%) and male workers. Another strength is a 75% response rate at baseline, of which 96% of participants granted access to their medical-administrative data. In addition, our data allowed the development of a gender measure covering all four recommended gender dimensions. Considering the state-of-knowledge of our research field, this is a milestone. Also, most gendered and CVD risk factors variables were measured using validated questionnaires and cases of CVD were identified using validated algorithms for medical-administrative databases. A further strength is the consideration of several covariates, including age and CVD risk factors, which were adjusted in the models. The main results were also stratified by sex, uncovering important differences for the first time.

The present study also has limitations. First, one could question the biological plausibility that such a gender measure would contribute to increase participants’ CVD risk over decades. Based on a life course approach [59], biological, behavioral, and social pathways operate across the life course, as well as across generations, to link previous exposures and experiences to later-life health and thus influence the development of chronic diseases [60]. These pathways typically include sociocultural and environmental factors [51, 59], such as those included in the proposed gender measure. Second, there is no official definition for variables related to gender in the literature yet. The presented gender measure was developed using data first collected approximately 30 years ago, and gender has been evolving since then. Nevertheless, within the PROQ-Study cohort, the prevalence rates of most variables related to gender were stable across sexes from baseline (1991–1993) to both 8-year (1999–2001) and 24-year (2015–2018) follow-ups, except for a decrease in the prevalence of participants with high family load at the latest follow-up, as well as an increased number of females with university education and professional positions at that time point. Those changes were expected, as there was only a small number of participants with children aged < 18 living with them at the 24-year follow-up, and a societal tendency among Canadian females to pursue education and subsequently hold higher occupational positions in the past decades [61, 62] (Tables S8–10). The temporal validity of our gender measure was also supported by a complementary analysis showing similar findings when the gender measure was recreated at the 8-year follow-up (1999–2001) (Table S11). However, and most importantly, given the complexity of societal evolution and the multifaceted nature of gender, certain variables related to gender (e.g., education and personality traits) might vary across and within cultures and change over time. Therefore, these variables would benefit from being tailored and adapted to local realities. Although our study introduced a new gender measure based on several variables embedded within four interrelated dimensions, it is plausible that other variables or dimensions of gender remain uncovered, necessitating further research efforts. The exportability of the method used in the present study to select and combine variables related to gender enables researchers to recreate their own specific gender measures, adapted to their culture and context. Third, in our approach, variables related to gender were dichotomized into binary categories of characteristics traditionally ascribed to either men or women to compute the gender score (as described in step 3). This binary categorization may have led to an underestimation of gender diversity within the gender measure. Fourth, the external validity of the study population is limited, as our sample was made up of white-collar workers. However, female and male participants held a wide variety of white-collar jobs, which ensures diversity [21]. White-collar workers constitute the largest segment of the Canadian workforce, which supports the scope of the results. We therefore recommend future studies to examine the validity of the variables related to gender across cultures, as well as within a wide range of occupational settings. Fifth, despite the advantages of using this gender measure when we do not have a gender measure, potential inherent pitfalls should be acknowledged. For example, such a gender measure does not replace an original one obtained through primary data collection, especially as it does not capture how participants perceive their own gender identity. The fact that our measure combines several characteristics related to gender makes it difficult to pinpoint underlying explanations behind the differences observed. Using this measure to uncover gender differences should therefore be approached as an exploratory step toward more extensive examinations. Finally, to gain a more comprehensive understanding of sex and gender disparities in cardiovascular health, future research endeavors should explore additional outcomes in the continuum of cardiovascular alterations. These may range from earlier-stage conditions like hypertension and arterial stiffness to more severe ones such as heart failure, and severe arrhythmias.

## Conclusions

A new gender measure was developed and linked to the 28-year incidence of CVD. In our cohort, both sex and gender were associated with CVD incidence. These findings suggest disparities related to sex and gender in cardiovascular health, reiterating the importance of considering both factors as partly independent predictors of CVD. Moving forward, integrating both sex and gender measures into clinical and population health research may enhance the inclusivity of prevention strategies. To facilitate this, future studies that utilize secondary data might find value in applying our exportable methodology to construct gender measures tailored to their specific cultures and contexts.

## Supplementary Information

Additional File 1: Tables S1-S11. Table S1- Participant characteristics according the to sex, at baseline. Table S2- Associations between female sex at baseline and the 28-year CVD incidence. Table S3- Associations between gender tertiles at baseline and the 28-year CVD incidence after excluding psychosocial stressors at work and working hours, stratified by sex. Table S4- Associations between gender tertiles at baseline and the 28-year CVD incidence after excluding personality traits, stratified by sex. Table S5- Associations between gender-related variables at baseline and the 28-year CVD incidence, stratified by sex. Table S6- Associations between gender score across sextiles at baseline and the 28-year CVD incidence, stratified by sex. Table S7- Associations between gender tertiles at baseline and the 28-year CVD incidence, including all 16 gender-related variables, stratified by sex. Table S8- Frequency of the 16 gender-related variables for males and females. Table S9- Frequency of the 16 gender-related variables for males and females. Table S10- Frequency of the 16 gender-related variables for males and females. Table S11- Associations between the gender-related variables at the 8-year follow-up and sex, modelling the probability of being a female.

## Data Availability

Please send all requests for study data or materials to Mahée Gilbert-Ouimet (mahee_gilbert-ouimet@uqar.ca). Data underlying the findings described in their manuscript cannot be freely available to other researchers since all data related to cardiovascular diseases cannot be made available because they are Quebec medicoadministrative data. Access to these data requires both the consent of the participants and approval from the Commission d'accès à l'information (CAI) for each specific project wishing to use them. Access is granted only to the researchers directly responsible for the approved projects. Conditions for usage of this data are presented here: https://www.cai.gouv.qc.ca/

## Abbreviations

CVD: Cardiovascular diseases
WHO: World Health Organization
PROQ-Study: PROspective Quebec Study
IHD: Ischemic heart disease
ICD: International Classification of Disease
CCP: Canadian Classification of Diagnostic, Therapeutic, and Surgical Procedures
CCI: Canadian Classification of Health Interventions
BMI: Body mass index
WHR: Waist-hip ratio
OR: Odds ratio
HR: Hazard ratio
CI: Confidence interval
SD: Standard deviation
