# Supplementary material for: Developing a gender measure and examining its association with cardiovascular diseases incidence: a 28-year prospective cohort study
Source: BMC Med. 2024 Oct 29;22:498. doi: 10.1186/s12916-024-03706-3 (PMC11520886; doi:10.1186/s12916-024-03706-3)
Supplement: Supplementary file 1 — Additional File 1: Tables S1-S11. Table S1- Participant characteristics according the to sex, at baseline. Table S2- Associations between female sex at baseline and the 28-year CVD incidence. Table S3- Associations between gender tertiles at baseline and the 28-year CVD incidence after excluding psychosocial stressors at work and working hours, stratified by sex. Table S4- Associations between gender tertiles at baseline and the 28-year CVD incidence after excluding personality traits, stratified by sex. Table S5- Associations between gender-related variables at baseline and the 28-year CVD incidence, stratified by sex. Table S6- Associations between gender score across sextiles at baseline and the 28-year CVD incidence, stratified by sex. Table S7- Associations between gender tertiles at baseline and the 28-year CVD incidence, including all 16 gender-related variables, stratified by sex. Table S8- Frequency of the 16 gender-related variables for males and females. Table S9- Frequency of the 16 gender-related variables for males and females. Table S10- Frequency of the 16 gender-related variables for males and females. Table S11- Associations between the gender-related variables at the 8-year follow-up and sex, modelling the probability of being a female. [file 12916_2024_3706_MOESM1_ESM.docx]

**Additional File 1**

**Table S1.** Participant characteristics according the to sex, at baseline (1991-93)

|  | Males (N=4,243) | Females (N=4,186) | *p** |
| --- | --- | --- | --- |
| N (%) or Mean (SD) | | |  |
| **Demographic characteristics** |  |  |  |
| Age, Years, Mean (SD) | 41.6 (8.8) | 38.2 (7.9) | <.0001 |
| Married/in a relationship | 3,338 (78.7) | 2,844 (67.9) | <.0001 |
| **CVD risk factors**** |  |  |  |
| Obesity | 456 (10.8) | 315 (7.6) | <.0001 |
| High risk WHR | 2,759 (65.3) | 315 (7.7) | <.0001 |
| Current smoking | 869 (20.7) | 1,078 (26.0) | <.0001 |
| High risk alcohol consumption | 249 (5.9) | 122 (2.9) | <.0001 |
| Low physical activity | 1,566 (37.0) | 1,952 (46.8) | <.0001 |
| Family history of CVD | 1,239 (29.5) | 1,340 (32.4) | 0.0041 |
| Diabetes | 92 (2.2) | 79 (1.9) | 0.3571 |
| Cholesterol | 892 (21.2) | 419 (10.1) | <.0001 |
| Hypertension status | 755 (17.8) | 258 (6.2) | <.0001 |
| Antihypertensive medication | 134 (3.2) | 97 (2.3) | 0.0177 |
| Menopause*** | - | 365 (8.7) | - |
| Use of oral contraceptives*** | - | 3,441(83.0) | - |
| Hormone replacement therapy*** | - | 402 (9.7) | - |

* *p* for Student’s t-test (for age continuous) and Chi-square test (for all other categorical variables)

**Missing values are present for these different variables

***Only for females

**Table S2.** Associations between female sex at baseline (1991-93) and the 28-year CVD incidence (N=3,865)

| **Sex** | **HR (95% CI)*** | **HR (95% CI)**** |
| --- | --- | --- |
| Male | 1·00 (-) | 1·00 (-) |
|  |  |  |
| Female | **0.50 (0.44-0.56)** | **0.65 (0.56-0.77)** |

Bold values denote statistical significance (*p*<0.05).

*Age adjusted through time scale and further adjusted for menopausal status, use of oral contraceptives and hormone replacement therapy at baseline. N=3,811, because of missing values for these different variables.

** Further adjusted for BMI (kg/m2): BMI (underweight/normal (<25), overweight (25-29.9), and 3) obesity (≥30)); WHR: 1) low risk (<0.90 in men and <0.85 in women), and 2) high risk (≥0.90 in men and ≥0.85 in women); smoking: 1) non-smoker, 2) former smoker, and 3) current smoker (occasional + regular)); alcohol consumption: 1) abstinent (0 consumption/week), 2) low risk (≤15 consumptions/week for men and ≤10 for women), and 3) high risk (>15 consumptions/week for men and >10 for women)); physical activity: 1) active (≥3 weekly), 2) moderate (1 or 2 weekly), and 3) low (<1 weekly)); family history of CVD: 1) no, and 2) yes), diabetes 1) no, and 2) yes; cholesterol: 1) no, and 2) yes; hypertension status: a person was considered hypertensive if they had a blood pressure (SBP) ≥140 and/or diastolic blood pressure (DBP) ≥90; 1) no, and 2) yes; antihypertensive medication 1) no, and 2) yes) at baseline. N=3,778 for males and N=3,583 for females because of missing values for these different variables.

**Table S3.** Associations between gender tertiles at baseline (1991-93) and the 28-year CVD incidence after excluding psychosocial stressors at work and working hours, stratified by sex (N=7,821)

|  | **Number of participants** | **Number of events** | **HR (95% CI)*** | **HR (95% CI)**** |
| --- | --- | --- | --- | --- |
| **Males** | 3,956 | 1,086 |  |  |
| Tertile 1 | 2,171 | 634 | 1·00 (-) | 1·00 (-) |
| Tertile 2 | 1,388 | 323 | 0.95 (0.83-1.09) | 0.95 (0.82-1.09) |
| Tertile 3 | 397 | 129 | **1.37 (1.13-1.66)** | **1.30 (1.06-1.58)** |
| **Females** | 3,865 | 519 |  |  |
| Tertile 1 | 481 | 66 | 1·00 (-) | 1·00 (-) |
| Tertile 2 | 1,248 | 159 | 0.99 (0.74-1.32) | 0.88 (0.65-1.19) |
| Tertile 3 | 2,136 | 294 | 0.96 (0.73-1.25) | 0.80 (0.61-1.06) |

Bold values denote statistical significance (*p*<0.05).

*Age adjusted through time scale and further adjusted for menopausal status, use of oral contraceptives and hormone replacement therapy at baseline for females. N=3,811 for females because of missing values for these different variables.

** Further adjusted for BMI (kg/m2): BMI (underweight/normal (<25), overweight (25-29.9), and 3) obesity (≥30)); WHR: 1) low risk (<0.90 in men and <0.85 in women), and 2) high risk (≥0.90 in men and ≥0.85 in women); smoking: 1) non-smoker, 2) former smoker, and 3) current smoker (occasional + regular)); alcohol consumption: 1) abstinent (0 consumption/week), 2) low risk (≤15 consumptions/week for men and ≤10 for women), and 3) high risk (>15 consumptions/week for men and >10 for women)); physical activity: 1) active (≥3 weekly), 2) moderate (1 or 2 weekly), and 3) low (<1 weekly)); family history of CVD: 1) no, and 2) yes), diabetes 1) no, and 2) yes; cholesterol: 1) no, and 2) yes; hypertension status: a person was considered hypertensive if they had a blood pressure (SBP) ≥140 and/or diastolic blood pressure (DBP) ≥90; 1) no, and 2) yes; antihypertensive medication 1) no, and 2) yes) at baseline. N=3,778 for males and N=3,583 for females because of missing values for these different variables.

**Table S4.** Associations between gender tertiles at baseline (1991-93) and the 28-year CVD incidence after excluding personality traits, stratified by sex (N=7,821)

|  | **Number of participants** | **Number of events** | **HR (95% CI)*** | **HR (95% CI)**** |
| --- | --- | --- | --- | --- |
| **Males** | 3,956 | 1,086 |  |  |
| Tertile 1 | 2,111 | 598 | 1·00 (-) | 1·00 (-) |
| Tertile 2 | 1,437 | 340 | 1.03 (0.90-1.18) | 1.00 (0.87-1.15) |
| Tertile 3 | 408 | 148 | **1.57 (1.31-1.88)** | **1.42 (1.17-1.72)** |
| **Females** | 3,865 | 519 |  |  |
| Tertile 1 | 531 | 68 | 1·00 (-) | 1·00 (-) |
| Tertile 2 | 1,204 | 148 | 0.95 (0.71-1.27) | 0.91 (0.67-1.23) |
| Tertile 3 | 2,130 | 303 | 1.00 (0.77-1.30) | 0.89 (0.67-1.17) |

Bold values denote statistical significance (*p*<0.05).

*Age adjusted through time scale and further adjusted for menopausal status, use of oral contraceptives and hormone replacement therapy at baseline for females. N=3,811 for females because of missing values for these different variables.

** Further adjusted for BMI (kg/m2): BMI (underweight/normal (<25), overweight (25-29.9), and 3) obesity (≥30)); WHR: 1) low risk (<0.90 in men and <0.85 in women), and 2) high risk (≥0.90 in men and ≥0.85 in women); smoking: 1) non-smoker, 2) former smoker, and 3) current smoker (occasional + regular)); alcohol consumption: 1) abstinent (0 consumption/week), 2) low risk (≤15 consumptions/week for men and ≤10 for women), and 3) high risk (>15 consumptions/week for men and >10 for women)); physical activity: 1) active (≥3 weekly), 2) moderate (1 or 2 weekly), and 3) low (<1 weekly)); family history of CVD: 1) no, and 2) yes), diabetes 1) no, and 2) yes; cholesterol: 1) no, and 2) yes; hypertension status: a person was considered hypertensive if they had a blood pressure (SBP) ≥140 and/or diastolic blood pressure (DBP) ≥90; 1) no, and 2) yes; antihypertensive medication 1) no, and 2) yes) at baseline. N=3,778 for males and N=3,583 for females because of missing values for these different variables.

**Table S5.** Associations between gender-related variables at baseline (1991-93) and the 28-year CVD incidence, stratified by sex (N=7,821)

| **Gender-related variables** |  | **HR (95% CI)*** | ***p***** | **HR (95% CI)***** | ***p***** |
| --- | --- | --- | --- | --- | --- |
| **Males (N=3,956)** |  |  |  |  |  |
| Marital status | Single | (1·00) (-) | 0.4637 | (1·00) (-) | 0.3528 |
|  | Married/in a relationship | 1.06 (0.91-1.24) |  | 1.08(0.92-1.28) |  |
| Working hours in main paid job | 10-20 h/w | 0.43 (0.06-3.04) | 0.4555 | 0.45 (0.06-3.21) | 0.7195 |
|  | 21-34 h/w | 1.16 (0.87-1.53) |  | 1.01 (0.75-1.36) |  |
|  | 35-40 h/w | (1·00) (-) |  | (1·00) (-) |  |
|  | ≥41 h/w | 0.92 (0.77-1.11) |  | 0.92 (0.76-1.11) |  |
| Hours working overtime | None | (1·00) (-) | 0.1787 | (1·00) (-) | 0.5487 |
|  | <10 h/w | 1.04 (0.89-1.21) |  | 1.09 (0.92-1.28) |  |
|  | 10-20 h/w | 0.98 (0.76-1.26) |  | 0.96 (0.74-1.24) |  |
|  | ≥21 h/w | **1.63 (1.05-2.55)** |  | 1.29 (0.78-2.12) |  |
| Family load | Low | (1·00) (-) | 0.7670 | (1·00) (-) | 0.9552 |
|  | Moderate | 1.00 (0.86-1.16) |  | 1.02 (0.88-1.19) |  |
|  | High | 0.95 (0.82-1.11) |  | 1.01 (0.86-1.19) |  |
| Physical job demands | Unexposed | (1·00) (-) | 0.2235 | (1·00) (-) | 0.7560 |
|  | Low | 1.06 (0.93-1.20) |  | 1.01 (0.89-1.16) |  |
|  | Moderate | 1.24 (0.89-1.72) |  | 1.12 (0.80-1.58) |  |
|  | High | 2.03 (0.84-4.90) |  | 1.56 (0.58-4.21) |  |
| High psychological demands | Unexposed | (1·00) (-) | 0.1213 | (1·00) (-) | 0.1332 |
|  | Exposed | 1.10 (0.98-1.24) |  | 1.10 (0.97-1.25) |  |
| Low job control | Unexposed | (1·00) (-) | 0.0072 | (1·00) (-) | 0.0147 |
|  | Exposed | **1.18 (1.05-1.33)** |  | **1.17 (1.03-1.32)** |  |
| Anger-In | Low | (1·00) (-) | 0.0542 | (1·00) (-) | 0.0859 |
|  | Moderate | 0.99 (0.84-1.18) |  | 1.02 (0.85-1.21) |  |
|  | High | **1.18 (1.03-1.36)** |  | **1.17 (1.01-1.36)** |  |
| Anger-Out | Low | (1·00) (-) | 0.7724 | (1·00) (-) | 0.5748 |
|  | Moderate | 1.07 (0.92-1.24) |  | 1.04 (0.90-1.22) |  |
|  | High | 1.05 (0.89-1.25) |  | 1.01 (0.85-1.20) |  |
| Anger-Discuss | Low | (1·00) (-) | 0.5618 | (1·00) (-) | 0.7776 |
|  | Moderate | 0.95 (0.82-1.10) |  | 0.97 (0.83-1.12) |  |
|  | High | 1.05 (0.91-1.22) |  | 1.05 (0.90-1.23) |  |
| Cynicism | Low | (1·00) (-) | <.0001 | (1·00) (-) | 0.0021 |
|  | Moderate | **1.16 (1.01-1.33)** |  | 1.15 (1.00-1.32) |  |
|  | High | **1.40 (1.20-1.63)** |  | **1.32 (1.13-1.55)** |  |
| Low social support outside from work | Unexposed | (1·00) (-) | 0.9241 | (1·00) (-) | 0.7667 |
|  | Exposed | 1.01 (0.87-1.16) |  | 1.02 (0.88-1.18) |  |
| Low social support at work | Unexposed | (1·00) (-) | 0.1044 | (1·00) (-) | 0.5765 |
|  | Exposed | 1.10 (0.98-1.25) |  | 1.04 (0.91-1.18) |  |
| Level of education | Secondary | (1·00) (-) | <.0001 | (1·00) (-) | <.0001 |
|  | College | **0.67 (0.56-0.80)** |  | **0.68 (0.57-0.82)** |  |
|  | University | **0.61 (0.52-0.71)** |  | **0.69 (0.59-0.81)** |  |
| Occupational position | Office workers | (1·00) (-) | 0.0004 | (1·00) (-) | 0.0255 |
|  | Technicians | 0.91 (0.73-1.13) |  | 0.93 (0.74-1.17) |  |
|  | Professional workers | **0.69 (0.57-0.84)** |  | **0.76 (0.62-0.94)** |  |
|  | Managers | **0.76 (0.61-0.94)** |  | **0.79 (0.63-1.00** |  |
|  | Others | 0.90 (0.62-1.31) |  | 1.02 (0.69-1.49) |  |
| **Females (N=3,865)** |  |  |  |  |  |
| Marital status | Single | (1·00) (-) | 0.4022 | (1·00) (-) | 0.3178 |
|  | Married/in a relationship | 1.08 (0.90-1.31) |  | 1.11 (0.91-1.35) |  |
| Working hours in main paid job | 10-20 h/w | 0.84 (0.31-2.25) | 0.9854 | 0.96 (0.36-2.58) | 0.9943 |
|  | 21-34 h/w | 0.97 (0.69-1.36) |  | 1.05 (0.73-1.50) |  |
|  | 35-40 h/w | (1·00) (-) |  | (1·00) (-) |  |
|  | ≥41 h/w | 1.00 (0.66-1.51) |  | 1.00 (0.65-1.54) |  |
| Hours working overtime | None | (1·00) (-) | 0.2357 | (1·00) (-) | 0.1605 |
|  | <10 h/w | 1.28 (0.99-1.64) |  | **1.31 (1.01-1.71)** |  |
|  | 10-20 h/w | 1.08 (0.72-1.62) |  | 0.97 (0.63-1.49) |  |
|  | ≥21 h/w | 1.35 (0.70-2.62) |  | 1.42 (0.73-2.77) |  |
| Family load | Low | (1·00) (-) | 0.2900 | (1·00) (-) | 0.1279 |
|  | Moderate | 1.06 (0.85-1.32) |  | 1.01 (0.80-1.28) |  |
|  | High | 1.19 (0.95-1.49) |  | 1.24 (0.98-1.56) |  |
| Physical job demands | Unexposed | (1·00) (-) | 0.2172 | (1·00) (-) | 0.3083 |
|  | Low | 0.99 (0.82-1.20) |  | 0.97 (0.80-1.18) |  |
|  | Moderate | 1.32 (0.72-2.41) |  | 1.43 (0.76-2.69) |  |
|  | High | 2.38 (0.97-5.81) |  | 1.97 (0.79-4.87) |  |
| High psychological demands | Unexposed | (1·00) (-) | 0.7845 | (1·00) (-) | 0.6869 |
|  | Exposed | 1.03 (0.86-1.23) |  | 1.04 (0.86-1.26) |  |
| Low job control | Unexposed | (1·00) (-) | 0.8727 | (1·00) (-) | 0.7510 |
|  | Exposed | 0.98 (0.81-1.20) |  | 0.97 (0.79-1.19) |  |
| Anger-In | Low | (1·00) (-) | 0.8471 | (1·00) (-) | 0.6433 |
|  | Moderate | 1.10 (0.87-1.39) |  | 1.14 (0.90-1.45) |  |
|  | High | 1.07 (0.87-1.32) |  | 1.07 (0.86-1.33) |  |
| Anger-Out | Low | (1·00) (-) | 0.4952 | (1·00) (-) | 0.3882 |
|  | Moderate | 0.95 (0.76-1.18) |  | 0.94 (0.74-1.18) |  |
|  | High | 1.18 (0.92-1.50) |  | 1.17 (0.91-1.52) |  |
| Anger-Discuss | Low | (1·00) (-) | 0.2792 | (1·00) (-) | 0.2894 |
|  | Moderate | 0.93 (0.73-1.19) |  | 0.94 (0.73-1.20) |  |
|  | High | 0.83 (0.66-1.04) |  | 0.81 (0.64-1.02) |  |
| Cynicism | Low | (1·00) (-) | 0.0256 | (1·00) (-) | 0.0573 |
|  | Moderate | 0.94 (0.77-1.15) |  | 0.92 (0.74-1.14) |  |
|  | High | **1.29 (1.03-1.61)** |  | 1.24 (0.98-1.56) |  |
| Hostility | Low | (1·00) (-) | 0.9489 | (1·00) (-) | 0.6418 |
|  | Moderate | 0.98 (0.77-1.25) |  | 0.89 (0.70-1.15) |  |
|  | High | 1.02 (0.82-1.27) |  | 0.91 (0.72-1.15) |  |
| Low social support outside from work | Unexposed | (1·00) (-) | 0.7995 | (1·00) (-) | 0.5523 |
|  | Exposed | 1.02 (0.86-1.22) |  | 1.06 (0.88-1.27) |  |
| Low social support at work | Unexposed | (1·00) (-) | 0.7000 | (1·00) (-) | 0.7003 |
|  | Exposed | 0.97 (0.81-1.15) |  | 0.96 (0.80-1.16) |  |
| Level of education | Secondary | (1·00) (-) | 0.2543 | (1·00) (-) | 0.8954 |
|  | College | 0.91 (0.74-1.12) |  | 1.04 (0.83-1.29) |  |
|  | University | 0.83 (0.66-1.04) |  | 0.97 (0.76-1.24) |  |
| Occupational position | Office workers | (1·00) (-) | 0.8694 | (1·00) (-) | 0.9660 |
|  | Technicians | 0.93 (0.73-1.19) |  | 0.94 (0.72-1.21) |  |
|  | Professional workers | 0.89 (0.70-1.13) |  | 1.03 (0.80-1.33) |  |
|  | Managers | 0.95 (0.62-1.47) |  | 1.01 (0.64-1.58) |  |
|  | Others | 1.07 (0.71-1.62) |  | 1.09 (0.71-1.67) |  |

Bold values denote statistical significance (*p*<0.05).

*Age adjusted through time scale and further adjusted for menopausal status, use of oral contraceptives and hormone replacement therapy at baseline for females. N=3,811 for females because of missing values for these different variables.

** *p* for global test using the Wald Chi-square statistic (Type 3 Analysis of Effects).

*** Further adjusted for CVD risk factors at baseline. N=3,778 for males and N=3,583 for females because of missing values for these different variables.

**Table S6.** Associations between gender score across sextiles at baseline (1991-93) and the 28-year CVD incidence, stratified by sex (N=7,821)

|  | **Number of participants** | **Number of events** | **HR (95% CI)*** | ***p****** | **HR (95% CI)**** | ***p****** |
| --- | --- | --- | --- | --- | --- | --- |
| **Males** | 3,956 | 1,086 |  |  |  |  |
| Sextile 1 (Masculine) | 1,172 | 358 | 1·00 (-) | 0.0019 | 1·00 (-) | 0.0178 |
| Sextile 2 (Masculine) | 1,010 | 265 | 0.97 (0.82-1.13) |  | 0.98 (0.83-1.16) |  |
| Sextile 3 (Androgynous) | 819 | 184 | 0.91 (0.76-1.09) |  | 0.97 (0.81-1.17) |  |
| Sextile 4 (Androgynous) | 572 | 148 | 1.07 (0.89-1.30) |  | 1·01 (0.83-1.24) |  |
| Sextile 5 (Feminine) | 274 | 97 | **1.56 (1.24-1.95)** |  | **1.48 (1.16-1.89)** |  |
| Sextile 6 (Feminine) | 109 | 34 | 1.29 (0.90-1.83) |  | 1.23 (0.84-1.78) |  |
| **Females** | 3,865 | 519 |  |  |  |  |
| Sextile 1 (Masculine) | 150 | 23 | 1·00 (-) | 0.6330 | 1·00 (-) | 0.1382 |
| Sextile 2 (Masculine) | 316 | 44 | 1.09 (0.65-1.80) |  | 0.99 (0.59-1.66) |  |
| Sextile 3 (Androgynous) | 494 | 57 | 0.96 (0.59-1.57) |  | 0.87 (0.53-1.42) |  |
| Sextile 4 (Androgynous) | 736 | 99 | 1.01 (0.64-1.60) |  | 0.92 (0.58-1.47) |  |
| Sextile 5 (Feminine) | 1,008 | 144 | 0.95 (0.61-1.48) |  | 0.78 (0.50-1.23) |  |
| Sextile 6 (Feminine) | 1,161 | 152 | 0.96 (0.61-1.49) |  | 0.79 (0.50-1.24) |  |

Bold values denote statistical significance (*p*<0.05).

*Age adjusted through time scale and further adjusted for menopausal status, use of oral contraceptives and hormone replacement therapy at baseline for females. N=3,811 for females because of missing values for these different variables.

** Further adjusted for BMI (kg/m2): BMI (underweight/normal (<25), overweight (25-29.9), and 3) obesity (≥30)); WHR: 1) low risk (<0.90 in men and <0.85 in women), and 2) high risk (≥0.90 in men and ≥0.85 in women); smoking: 1) non-smoker, 2) former smoker, and 3) current smoker (occasional + regular)); alcohol consumption: 1) abstinent (0 consumption/week), 2) low risk (≤15 consumptions/week for men and ≤10 for women), and 3) high risk (>15 consumptions/week for men and >10 for women)); physical activity: 1) active (≥3 weekly), 2) moderate (1 or 2 weekly), and 3) low (<1 weekly)); family history of CVD: 1) no, and 2) yes), diabetes 1) no, and 2) yes; cholesterol: 1) no, and 2) yes; hypertension status: a person was considered hypertensive if they had a blood pressure (SBP) ≥140 and/or diastolic blood pressure (DBP) ≥90; 1) no, and 2) yes; antihypertensive medication 1) no, and 2) yes) at baseline. N=3,778 for males and N=3,583 for females because of missing values for these different variables.

*p**** of the contrast for linear trend

**Table S7.** Associations between gender tertiles at baseline (1991-93) and the 28-year CVD incidence, including all 16 gender-related variables, stratified by sex (N=7,821)

|  | **Number of participants** | **Number of events** | **HR (95% CI)*** | **HR (95% CI)**** |
| --- | --- | --- | --- | --- |
| **Males** | 3,956 | 1,086 |  |  |
| Tertile 1 | 2,184 | 624 | 1·00 (-) | 1·00 (-) |
| Tertile 2 | 1,388 | 332 | 0.99 (0.87-1.13) | 0.99 (0.86-1.14) |
| Tertile 3 | 384 | 130 | **1.47 (1.22-1.78)** | **1.38 (1.13-1.69)** |
| **Females** | 3,865 | 519 |  |  |
| Tertile 1 | 465 | 66 | 1·00 (-) | 1·00 (-) |
| Tertile 2 | 1,232 | 158 | 0.97 (0.72-1.29) | 0.93 (0.69-1.26) |
| Tertile 3 | 2,168 | 295 | 0.92 (0.71-1.21) | 0.81 (0.61-1.08) |

Bold values denote statistical significance (*p*<0.05).

*Age adjusted through time scale and further adjusted for menopausal status, use of oral contraceptives and hormone replacement therapy at baseline for females. N=3,811 for females because of missing values for these different variables.

** Further adjusted for BMI (kg/m2): BMI (underweight/normal (<25), overweight (25-29.9), and 3) obesity (≥30)); WHR: 1) low risk (<0.90 in men and <0.85 in women), and 2) high risk (≥0.90 in men and ≥0.85 in women); smoking: 1) non-smoker, 2) former smoker, and 3) current smoker (occasional + regular)); alcohol consumption: 1) abstinent (0 consumption/week), 2) low risk (≤15 consumptions/week for men and ≤10 for women), and 3) high risk (>15 consumptions/week for men and >10 for women)); physical activity: 1) active (≥3 weekly), 2) moderate (1 or 2 weekly), and 3) low (<1 weekly)); family history of CVD: 1) no, and 2) yes), diabetes 1) no, and 2) yes; cholesterol: 1) no, and 2) yes; hypertension status: a person was considered hypertensive if they had a blood pressure (SBP) ≥140 and/or diastolic blood pressure (DBP) ≥90; 1) no, and 2) yes; antihypertensive medication 1) no, and 2) yes) at baseline. N=3,778 for males and N=3,583 for females because of missing values for these different variables.

**Table S8.** Frequency of the 16 gender-related variables for males and females (N=8429), 1991-1993

| **Gender-related variables** |  | **Males** | **Females** |
| --- | --- | --- | --- |
| Marital status | Single | 905 (21.3) | 1342 (32.1)  2,844 (67.9) |
|  | Married/in a relationship | 3,338 (78.7) |  |
| Working hours in main paid job | 10-20 h/w | 11(0.3) | 34 (0.8)  299 (7.1)  3681 (88)  172 (4.1) |
|  | 21-34 h/w | 140 (3.3) |  |
|  | 35-40 h/w | 3607 (85) |  |
|  | ≥41 h/w | 485 (11.4) |  |
| Hours working overtime | None | 3117 (73.5) | 3330 (79.5)  562 (13.4)  232 (5.6)  62 (1.5) |
|  | <10 h/w | 782 (18.4) |  |
|  | 10-20 h/w | 284 (6.7) |  |
|  | ≥21 h/w | 60 (1.4) |  |
| Family load | Low | 1273 (30) | 1360 (32.5)  1343 (32.1)  1483 (35.4) |
|  | Moderate | 1555 (36.7) |  |
|  | High | 1415 (33.3) |  |
| Physical job demands | Unexposed | 2743 (64.7) | 2670 (63.8)  1424 (34)  78 (1.9)  14 (0.3) |
|  | Low | 1353 (31.9) |  |
|  | Moderate | 132 (3.1) |  |
|  | High | 15 (0.3) |  |
| High psychological demands | Unexposed | 2345 (55.2) | 2570 (61.4)  1616 (38.6) |
|  | Exposed | 1898 (44.8) |  |
| Low job control | Unexposed | 1963 (46.3) | 1130 (27)  3056 (73) |
|  | Exposed | 2280 (53.7) |  |
| Anger-In | Low | 1259 (29.7) | 1581 (37.8)  1010 (24.1)  1464 (35) |
|  | Moderate | 1002 (23.6) |  |
|  | High | 1804 (42.5) |  |
| Anger-Out | Low | 2454 (57.9) | 2341 (56)  1052 (25.1)  662 (15.8) |
|  | Moderate | 969 (22.8) |  |
|  | High | 654 (15.4) |  |
| Anger-Discuss | Low | 1590 (37.5) | 850 (20.3)  1240 (29.6)  2000 (47.8) |
|  | Moderate | 1342 (31.6) |  |
|  | High | 1172 (27.6) |  |
| Cynicism | Low | 1999 (47.1) | 1963 (46.9)  1415 (33.8)  808 (19.3) |
|  | Moderate | 1399 (33) |  |
|  | High | 845 (20) |  |
| Hostility | Low | 1201 (28.3) | 1055 (25.2) |
|  | Moderate | 1297 (30.6) | 1273 (30.4) |
|  | High | 1745 (41.1) | 1858 (44.4) |
| Low social support outside from work | Unexposed | 1120 (26.4) | 1945 (46.5)  2241 (53.5) |
|  | Exposed | 3123 (73.6) |  |
| Low social support at work | Unexposed | 2542 (60) | 2117 (50.6)  2069 (49.4) |
|  | Exposed | 1701 (40) |  |
| Level of education | Secondary | 603 (14.2) | 1852 (44.2)  1272 (30.4)  1062 (25.4) |
|  | College | 1111 (26.2) |  |
|  | University | 2529 (59.6) |  |
| Occupational position | Office workers | 448 (10.5) | 2246 (53.7)  793 (19)  859 (20.5)  148 (3.5)  140 (3.3) |
|  | Technicians | 890 (21) |  |
|  | Professional workers | 2056 (48.5) |  |
|  | Managers | 736 (17.3) |  |
|  | Others | 113 (2.7) |  |

**Table S9.** Frequency of the 16 gender-related variables for males and females (N=6223), 1999-2001

| **Gender-related variables** |  | **Males** | **Females** |
| --- | --- | --- | --- |
| Marital status | Single | 595 ( 19.5) | 918 (29)  2248 (71) |
|  | Married/in a relationship | 2462 ( 80.5) |  |
| Working hours in main paid job | 10-20 h/w | 31 (1) | 36 (1.1)  420 (13.3)  2610 (82.4)  100 (3.2) |
|  | 21-34 h/w | 116 ( 3.8) |  |
|  | 35-40 h/w | 2622 ( 85.8) |  |
|  | ≥41 h/w | 288 ( 9.4) |  |
| Hours working overtime | None | 2618 (85.6) | 2812 (88.8)  164 (5.2)  51 (1.6)  139 (4.4) |
|  | <10 h/w | 241 (7.9) |  |
|  | 10-20 h/w | 87 (2.8) |  |
|  | ≥21 h/w | 111 (3.6) |  |
| Family load | Low | 1686 (55.2) | 1715 (54.2)  893 (28.2)  558 (17.6) |
|  | Moderate | 777 (25.4) |  |
|  | High | 594 (19.4) |  |
| Physical job demands | Unexposed | 2047 (67) | 2057 (65)  1059 (33.4)  43 (1.4)  7 (0.2) |
|  | Low | 900 (29.4) |  |
|  | Moderate | 90 (2.9) |  |
|  | High | 20 (0.6) |  |
| High psychological demands | Unexposed | 1552 (50.8) | 1797 (56.8)  1369 (43.2) |
|  | Exposed | 1505 (49.2) |  |
| Low job control | Unexposed | 1702 (55.7) | 1132 (35.7)  2034 (64.2) |
|  | Exposed | 1355 (44.3) |  |
| Anger-In | Low | 880 (28.8) | 1173 (37)  817 (25.8)  1176 (37.2) |
|  | Moderate | 778 (25.4) |  |
|  | High | 1399 (45.8) |  |
| Anger-Out | Low | 2062 (67.5) | 1983 (62.6)  726 (23)  457 (14.4) |
|  | Moderate | 588 (19.2) |  |
|  | High | 407 (13.3) |  |
| Anger-Discuss | Low | 1328 (43.4) | 709 (22.4)  1099 (34.7)  1358 (42.9) |
|  | Moderate | 1050 (34.4) |  |
|  | High | 679 (22.2) |  |
| Cynicism | Low | 1689 (55.2) | 1735 (54.8)  913 (28.8)  518 (16.4) |
|  | Moderate | 812 (26.6) |  |
|  | High | 556 (18.2) |  |
| Hostility | Low | 1025 (33.5) | 888 (28) |
|  | Moderate | 897 (29.3) | 952 (30.1) |
|  | High | 1135 (37.1) | 1326 (41.9) |
| Low social support outside from work | Unexposed | 727 (23.8) | 1467 (46.3)  1699 (53.7) |
|  | Exposed | 2330 (76.2) |  |
| Low social support from supervisor at work | Unexposed | 1573 (51.5) | 1543 (48.7)  1623 (51.3) |
|  | Exposed | 1484 (48.5) |  |
| Low social support from colleagues at work | Unexposed  Exposed | 1475 (48.2)  1582 (51.8) | 1564 (49.4)  1602 (50.6) |
|  |  |  |  |
| Level of education | Secondary | 359 (11.7) | 1246 (39.4)  935 (29.5)  985 (31.1) |
|  | College | 779 (25.5) |  |
|  | University | 1919 (62.8) |  |
| Occupational position | Office workers | 241 (7.9) | 1330 (42)  842 (26.6)  762 (24.1)  139 (4.4)  93 (2.9) |
|  | Technicians | 655 (21.4) |  |
|  | Professional workers | 1615 (52.8) |  |
|  | Managers | 479 (15.7) |  |
|  | Others | 67 (2.2) |  |

**Table S10.** Frequency of the 16 gender-related variables for males and females (N=2087), 2015-2018

| **Gender-related variables** |  | **Males** | **Females** |
| --- | --- | --- | --- |
| Marital status | Single | 223 (21.5) | 333 (32.2)  702 (67.8) |
|  | Married/in a relationship | 816 (78.5) |  |
| Working hours in all jobs combined | 10-20 h/w | 217 (21) | 163 (16.1)  168 (16.6)  616 (61)  63 (6.3) |
|  | 21-34 h/w | 142 (13.8) |  |
|  | 35-40 h/w | 576 (55.9) |  |
|  | ≥41 h/w | 96 (9.3) |  |
| Family load | Low | 890 (85) | 926 (89.4)  105 (10.1)  5 (0.5) |
|  | Moderate | 151 (14.4) |  |
|  | High | 6 (0.6) |  |
| Physical job demands (handling loads) | Unexposed | 775 (76.4) | 825 (82.6)  134 (13.4)  28 (2.8)  12 (1.2) |
|  | Low | 168 (16.6) |  |
|  | Moderate | 53 (5.2) |  |
|  | High | 18 (1.8) |  |
| Physical job demands (staying in a painful or tiring posture for a long time) | Unexposed | 749 (74) | 705 (70.9) |
|  | Low | 202 (19.9) | 159 (16) |
|  | Moderate | 48 (4.7) | 91 (9.1) |
|  | High | 14 (1.4) | 40 (4) |
| High psychological demands | Unexposed | 633 (62.8) | 592 ( 59.7)  400 (40.3) |
|  | Exposed | 375 (37.2) |  |
| Low job control | Unexposed | 579 (57.3) | 376 (37.8)  618 (62.1) |
|  | Exposed | 431 (42.7) |  |
| Anger-In | Low | 230 (29.8) | 227 (32.8)  183 (26.4)  282 (40.8) |
|  | Moderate | 170 (22) |  |
|  | High | 372 (48.2) |  |
| Anger-Out | Low | 604 (78.1) | 505 (72.7)  129 (18.6)  61 (8.8) |
|  | Moderate | 120 (15.5) |  |
|  | High | 49 (6.3) |  |
| Anger-Discuss | Low | 385 (49.9) | 188 (27.3)  239 (34.6)  263 (38.1) |
|  | Moderate | 255 (33) |  |
|  | High | 132 (17.1) |  |
| Cynicism | Low | 468 (60.5) | 396 (57.2)  199 (28.7)  98 (14.1) |
|  | Moderate | 200 (25.8) |  |
|  | High | 106 (13.7) |  |
| Hostility | Low | 322 (41.7) | 225 (32.5) |
|  | Moderate | 216 (27.9) | 221 (31.9) |
|  | High | 235 (30.4) | 247 ( 35.6) |
| Low social support outside from work | Unexposed | 227 ( 22.1) | 403 (40.1)  603 (59.9) |
|  | Exposed | 802 (77.9) |  |
| Low social support from supervisor at work | Unexposed | 483 (59.8) | 516 (57.9)  375 (42.1) |
|  | Exposed | 325 (40.2) |  |
| Low social support from colleagues at work | Unexposed | 567 (58.3) | 541 (56.2) |
|  | Exposed | 405 (41.7) | 422 (43.8) |
| Level of education | Secondary | 89 (8.5) | 255 (24.6)  339 (32.7)  443 (42.7) |
|  | College | 268 (25.6) |  |
|  | University | 690 (65.9) |  |
| Occupational position | Office workers | 25 (2.5) | 186 (18.4)  158 (15.6)  417 (41.3)  104 (10.3)  146 (14.4) |
|  | Technicians | 74 (7.5) |  |
|  | Professional workers | 519 (52.6) |  |
|  | Managers | 203 (20.5) |  |
|  | Others | 166 (16.8) |  |

**Table S11.** Associations between the gender-related variables at the 8-year follow-up (1999-2001) and sex, modelling the probability of being a female (N= 6,223)

| **Gender-related variables** |  | **OR (95% CI)** | ***p**** |
| --- | --- | --- | --- |
| Marital status | Single | (1·00) (-) | <.0001 |
|  | Married/in a relationship | **0.63 (0.54-0.73)** |  |
| Working hours in main paid job | 10-20 h/w | 1.36 (0.74-2.48) | <.0001 |
|  | 21-34 h/w | **3.69 (2.89-4.71)** |  |
|  | 35-40 h/w | (1·00) (-) |  |
|  | ≥41 h/w | 0.88 (0.66-1.17) |  |
| Hours working overtime | None | (1·00) (-) | 0.0003 |
|  | <10 h/w | **0.66 (0.51-0.85)** |  |
|  | 10-20 h/w | 0.52 (0.34-0.80) |  |
|  | ≥21 h/w | 0.98 (0.72-1.34) |  |
| Family load | Low | (1·00) (-) | 0.0099 |
|  | Moderate | **1.25 (1.08-1.45)** |  |
|  | High | 1.11 (0.94-1.32) |  |
| Physical job demands | Unexposed | (1·00) (-) | <.0001 |
|  | Low | **0.96 (0.83-1.09)** |  |
|  | Moderate | **0.24 (0.15-0.37)** |  |
|  | High | **0.16 (0.06-0.40)** |  |
| High psychological demands | Unexposed | (1·00) (-) | <.0001 |
|  | Exposed | **1.52 (1.33-1.74)** |  |
| Low job control | Unexposed | (1·00) (-) | 0.0003 |
|  | Exposed | **1.28 (1.12-1.47)** |  |
| Anger-In | Low | (1·00) (-) | 0.0040 |
|  | Moderate | 0.85 (0.72-0.99) |  |
|  | High | **0.77 (0.66-0.90)** |  |
| Anger-Out | Low | (1·00) (-) | 0.0263 |
|  | Moderate | 1.23 (1.05-1.44) |  |
|  | High | 1.11 (0.92-1.34) |  |
| Anger-Discuss | Low | (1·00) (-) | <.0001 |
|  | Moderate | **1.55 (1.33-1.81)** |  |
|  | High | **2.68 (2.26-3.16)** |  |
| Cynicism | Low | (1·00) (-) | 0.0003 |
|  | Moderate | 1.01 (0.87-1.17) |  |
|  | High | **0.70 (0.59-0.84)** |  |
| Hostility | Low | (1·00) (-) | 0.0078 |
|  | Moderate | **1.21 (1.04-1.42)** |  |
|  | High | **1.27 (1.08-1.49)** |  |
| Low social support outside from work | Unexposed | (1·00) (-) | <.0001 |
|  | Exposed | **0.46 (0.40-0.52)** |  |
| Low social support at work | Unexposed | (1·00) (-) | 0.3281 |
|  | Exposed | 0.93 (0.82-1.06) |  |
| Level of education | Secondary | (1·00) (-) | <.0001 |
|  | College | **0.61 (0.51-0.74)** |  |
|  | University | **0.52 (0.42-0.64)** |  |
| Occupational position | Office workers | (1·00) (-) | <.0001 |
|  | Technicians | **0.25 (0.20-0.30)** |  |
|  | Professional workers | **0.10 (0.08-0.13)** |  |
|  | Managers | **0.07 (0.05-0.09)** |  |
|  | Others | **0.33 (0.22-0.49)** |  |

Bold values denote statistical significance (*p*<0.05).

* *p* for global test using the Wald Chi-square statistic (Type 3 Analysis of Effects)
